# Ensemble learning for detecting gene-gene interactions in colorectal cancer

**DOI:** 10.7717/peerj.5854

**Published:** 2018-10-29

**Authors:** Faramarz Dorani, Ting Hu, Michael O. Woods, Guangju Zhai

**Affiliations:** 1 Department of Computer Science, Memorial University, St. John’s, Newfoundland and Labrador, Canada; 2 Faculty of Medicine, Memorial University, St. John’s, Newfoundland and Labrador, Canada

**Keywords:** Ensemble learning, Gene-gene interaction, Colorectal cancer, Random forests, Gradient boosting machine, Genetic marker discovery, Epistasis, Complex diseases

## Abstract

Colorectal cancer (CRC) has a high incident rate in both men and women and is affecting millions of people every year. Genome-wide association studies (GWAS) on CRC have successfully revealed common single-nucleotide polymorphisms (SNPs) associated with CRC risk. However, they can only explain a very limited fraction of the disease heritability. One reason may be the common uni-variable analyses in GWAS where genetic variants are examined one at a time. Given the complexity of cancers, the non-additive interaction effects among multiple genetic variants have a potential of explaining the missing heritability. In this study, we employed two powerful ensemble learning algorithms, random forests and gradient boosting machine (GBM), to search for SNPs that contribute to the disease risk through non-additive gene-gene interactions. We were able to find 44 possible susceptibility SNPs that were ranked most significant by both algorithms. Out of those 44 SNPs, 29 are in coding regions. The 29 genes include *ARRDC5*, *DCC*, *ALK*, and *ITGA1*, which have been found previously associated with CRC, and *E2F3* and *NID2*, which are potentially related to CRC since they have known associations with other types of cancer. We performed pairwise and three-way interaction analysis on the 44 SNPs using information theoretical techniques and found 17 pairwise (*p* < 0.02) and 16 three-way (*p* ≤ 0.001) interactions among them. Moreover, functional enrichment analysis suggested 16 functional terms or biological pathways that may help us better understand the etiology of the disease.

## Introduction

The goal of most genome-wide association studies (GWAS) is to examine common genetic variations across the entire human genome in order to identify single-nucleotide polymorphisms (SNPs) that are associated with diseases or phenotypic traits (McCarthy et al., 2008; Hindorff et al., 2009; Foulkes, 2009; Lek et al., 2016). GWAS survey the human genome for causal factors (Hirschhorn & Daly, 2005), and have successfully identified genetic variants that influence the risks of many complex diseases, including cardiovascular diseases (Mohlke, Boehnke & Abecasis, 2008; Nikpay et al., 2015), autoimmune diseases (Samani et al., 2007), and cancers (Easton & Eeles, 2008; Lettre & Rioux, 2008; Michailidou et al., 2013).

Various statistical and computational approaches can be used to identify genetic variants associated with human diseases. Those approaches can be divided into two categories: uni-variable and multi-variable analyses. In the uni-variable analysis, single-locus tests examine each SNP independently for its association with the phenotype. The effect size (or penetrance) of a variant is calculated and scored based on its significance of association with the disease phenotype. Many single-SNP-based methods were used for initial GWAS analyses, but had limited success in detecting genetic risk factors and explaining disease heritability (Moore & Williams, 2002; Balding, 2006; Szymczak et al., 2009; Manolio et al., 2009).

Recent research has shifted toward using or developing multi-variable approaches that examine interactions among genetic variants (Friedman, Hastie & Tibshirani, 2001; Balding, 2006; Zhang & Liu, 2007; Frazer et al., 2009; Han & Chen, 2011; Bush & Moore, 2012; Beam, Motsinger-Reif & Doyle, 2014; Jing & Shen, 2015; Sun et al., 2017). It is more plausible that interactions of multiple factors rather than individual genetic variants, can better explain susceptibility of complex diseases (Moore & Williams, 2009; Mackay & Moore, 2014). Multi-locus methods analyze combinations of SNPs and are able to capture interactions among multiple SNPs in GWAS data. However, they present significant statistical and computational challenges in terms of developing powerful methods to model non-linear, non-additive SNP interactions, selecting most relevant genetic variables, and interpreting discovered gene-gene interaction models (Moore & Ritchie, 2004).

Machine learning methods that are able to derive predictive models through training from historical data have seen increasing applications in finding genetic markers for GWAS (Szymczak et al., 2009; Okser et al., 2014; Libbrecht & Noble, 2015; Niel et al., 2015). For instance, the Ridge regression method was employed to identify a risk susceptibility SNP in rheumatoid arthritis near the *HLA*-B gene (Sun et al., 2009). D’Angelo, Rao & Gu (2009) combined the least absolute shrinkage and selection operator with the principal component analysis and detected two significant gene-gene interactions in rheumatoid arthritis. Although successful in characterizing gene-gene interactions, the common regression methods are not able to search for combinations of SNPs in high-dimensional and large-scale GWAS data (Szymczak et al., 2009).

Meanwhile, ensemble learning methods, especially the random forests algorithm, have been explored to search for and characterize combinatorial and non-linear interactions in microarray gene expression data (Huynh-Thu et al., 2010) and in GWAS data (Yoshida & Koike, 2011; Wright, Ziegler & König, 2016; Niel et al., 2018; Niel & Sinoquet, 2018). For instance, random forests were shown to be able to rank both main-effect and interacting SNPs using its Gini Index variable importance measure (Kim et al., 2009; Chen & Ishwaran, 2012), and to identify many known and several new susceptibility SNPs associated with human diseases (Tang et al., 2009; Wang et al., 2009). In a comprehensive comparative study (Olson et al., 2017), it was suggested that when applied to classification problems in bioinformatics data, tree-based ensemble learning methods outperform others, including support vector machine and naive Bayes methods. A total of 13 machine learning methods were investigated and applied to 165 benchmarking bioinformatics datasets. The comparison results showed that gradient tree boosting and subsequently random forests performed the best in terms of achieving the best cross-validation (CV) classification accuracy.

In this article, we investigated two ensemble learning algorithms, random forests and gradient boosting machine (GBM), to search for risk susceptibility SNPs associated with colorectal cancer (CRC). We used a CRC GWAS dataset collected from the Canadian province of Newfoundland and Labrador. Both algorithms were able to capture the non-linear gene-gene interactions associated with the disease status, and to rank SNPs based on their disease associations through either main effects or interaction effects. We identified 44 SNPs ranked as the most significant by both algorithms, including both known CRC related genes such as *DCC* and *ALK*, and SNPs that have not been found previously associated with CRC. We further performed a gene-gene interaction analysis on the 44 SNPs using an information gain method, and were able to validate significant pairwise and three-way synergistic interactions among them. The functional enrichment analysis on the 29 genes mapped from the 44 SNPs also suggested several significant biological pathways that may help explain the risk of CRC.

## Methodology

### CRC GWAS data and preprocessing

The colorectal (CRC) GWAS case-control data were collected from CRC patients and healthy individuals with matching age, gender, and geographical distributions within the province of Newfoundland and Labrador, Canada. The CRC Transdisciplinary (CORECT) consortium coordinated the genotyping of data. Genotyping was conducted using a custom Affymetrix genome-wide platform (the Axiom CORECT Set) on two physical genotyping chips (pegs) for two datasets with around 1.2 and 1.1 million SNPs (Schumacher et al., 2015). The first dataset has 1,236,084 SNPs and 696 samples with 200 cases and 496 controls and the genotyping rate was 0.997. In GWAS, genotyping rate is computed as the percentage of samples (including both cases and controls) that are successfully genotyped. The second dataset has 1,134,514 SNPs and 656 cases with a genotyping rate of 0.888. Using PLINK (Purcell et al., 2007), a tool for analyzing genetic data, we merged these two datasets based on their common SNPs and obtained a dataset of 265,195 SNPs and 1,152 unique samples. Among the samples, 656 were cases and 496 were controls.

Next, we performed per-sample and per-marker quality control steps and linkage disequilibrium (LD) pruning on the CRC data using PLINK. In the per-sample quality control, sex check was performed, sex chromosomes were excluded, and samples with more than 1% missing genotypes and outlier heterozygosity rate were removed. In the per-marker quality control, SNPs with missing call rates higher than 5%, with minor allele frequencies (MAFs) less than 5%, or with Hardy-Weinberg equilibrium values greater than 0.0001 were removed. Then, we pruned SNPs that were dependent on each other (correlation coefficient *r*^2^ > 0.6, LD window size 2,000). Missing genotypes were imputed using the most frequent alleles in the population. Last, the genotypes were re-coded using numerical values {0, 1, 2} with 0 standing for homozygous reference, 1 for heterozygous variant, and 2 for homozygous variant. After the steps of quality control and imputation, the final preprocessed and balanced dataset had 186,251 SNPs and 944 samples (472 being cases and 472 being controls).

The original data collection was approved by Memorial University Health Research Ethics Authority (HREA) with the approval number HIC 01.70. Our study was recognized by HREA as the use of secondary data which have already been collected and de-identified, and did not require a clearance.

### Feature selection

Most computational methods in informatics find it prohibiting to analyze high dimensional GWAS data (Moore, Asselbergs & Williams, 2010) due to the massive number of attributes, that is, hundreds of thousands of SNPs in the data. It is impossible for machine learning methods to detect interactions by enumerating all combinations of SNPs in a GWAS dataset. Furthermore, the existence of redundant and irrelevant attributes hinders machine learning methods to reveal actual gene-gene interactions in the data. These together set the stage for the necessity of dimensionality reduction or feature (attribute) selection for analyzing GWAS data (Moore, Asselbergs & Williams, 2010).

Feature selection is frequently used as a data filtering step in machine learning when the original data contain noisy or irrelevant features, or attributes, that could compromise the prediction power of learning algorithms (Yu & Liu, 2003). Feature selection methods choose only a subset of the most important features, and thus reduce the dimensionality of the data, speed up the learning process, simplify the learned model, and improve the prediction performance (Dash & Liu, 1997; Guyon & Elisseeff, 2003).

In our previous study (Dorani & Hu, 2018), six feature selection algorithms, including chi-square, logistic regression, odds ratio, and three Relief-based algorithms (ReliefF, Tuned ReliefF (TuRF), and Spatial Uniform ReliefF) were compared based on how they rank the most important SNP attributes that contribute to the phenotypic outcome through gene-gene interactions. We applied the feature selection methods to both simulated and real GWAS datasets and showed that Relief-based methods, specifically TuRF, performed the best in filtering SNPs associated with a disease through interaction effects. This observation was also supported by other independent studies (Beretta & Santaniello, 2011; Urbanowicz et al., 2018). Therefore, in this study, we used the TuRF feature selection method to reduce the CRC GWAS dataset to be of a manageable size such that it can be analyzed using the downstream classification algorithms in a reasonable time frame.

### Ensemble learning

Many preliminary GWAS employed uni-variable approaches where interactions between multiple variables could be overlooked (Moore & Ritchie, 2004). Moreover, the parametric linear statistical models look at multiple variables but have limitations for detecting non-linear interactions (Moore, Asselbergs & Williams, 2010). Thanks to the intrinsic multi-variable and non-linear properties, tree-based ensemble learning methods have been proved to be a powerful analysis tool for detecting interacting genes in GWAS (Moore, Asselbergs & Williams, 2010; Upstill-Goddard et al., 2012). They can be used to train highly accurate classifiers, as well as to discover new genetic markers by ranking genetic variables based on their importance in classification.

Ensemble learning methods use an aggregation of predictors known as base learners. To produce a final prediction, the predictions of the base learners are weighted and the overall predictions are decided through majority voting for classifications and averaging for regressions. It has been shown that ensemble learning methods are powerful at reducing variance and overfitting by utilizing a collection of diverse base learners such as classification and regression trees (CART) (Breiman, 1996; Dietterich, 2000). In this article, we adopted two tree-based ensemble learning algorithms, random forests and GBM, to explore their power in detecting non-linear gene-gene interactions for GWAS.

### Random forests

One popular ensemble learning method is *bagging* (short for bootstrap aggregating) that uses bootstrapped samples of the training data to train classification or regression models separately (Breiman, 1996). Bagging reduces the variance of an estimated prediction function, and works especially well for high-variance, low-bias procedures, such as trees (Friedman, Hastie & Tibshirani, 2001). The random forests algorithm is a special case of bagging where the variables are randomly selected to determine the optimal split at each node of the tree (Breiman, 2001). Random forests are shown to be a very powerful regression and classification method which utilizes a large collection of possibly uncorrelated decision trees (Breiman, 2001; Szymczak et al., 2009; Ziegler, DeStefano & König, 2007; Schwarz, Inke & Ziegler, 2010). Each tree is grown using the CART methodology (Breiman, 1996).

In random forests, *ntree* trees are grown independently using bootstrapped training samples. While constructing an individual tree, for each node, *mtry* (<*M*) predictor variables are randomly picked from the total *M* variables in the original data. Then the best variable/partition-point among the *mtry* variables is picked and used to split the node into two daughter nodes. Each tree grows to its maximum size, that is, when there is only one sample in the leaves. To make a classification for a new testing sample, each tree casts a vote for the predicted class and the majority vote will be the final prediction result. Intuitively, reducing *mtry* will reduce the correlation between any pair of trees in the ensemble. For classification, the default value of *mtry* is $\lfloor \sqrt M \rfloor$; however, the best value for this parameter will depend on the application problem, and it should be treated as a tuning parameter (Breiman, 2001; Friedman, Hastie & Tibshirani, 2001).

The random forests algorithm is effective in uncovering interactions among genes that do not exhibit strong main effects (Moore, Asselbergs & Williams, 2010). The algorithm has been employed in various studies to predict rheumatoid arthritis risks (Sun et al., 2007), to rank SNP predictors (Schwarz et al., 2007; Sun et al., 2008), and to detect the epistatic effects associated with human diseases (Garca-Magariños et al., 2009; Pan et al., 2014).

Goldstein et al. (2010) indicated that using the random forests algorithm with default settings of hyper-parameters would not yield optimal results for large GWAS datasets. In contrast, tuning the hyper-parameters, including *mtry* (number of random variables to make best split at each tree node) and *ntree* (number of trees), and using greater values, work well generally for large GWAS datasets.

In this study, we used a very fast implementation of random forests provided in an R package called “ranger” (Wright & Ziegler, 2017). The “ranger” package provides all functionalities similar to the RandomForest package in R with much greater speed. Therefore, we can use it for the GWAS datasets with a large number of SNPs. We performed parameter tuning on *mtry* and *ntree* in our implementation. For *mtry* we selected the values of {100, 200, 300, 500, 1,000}, and the selected values for *ntree* were {500, 1,000, 2,000}. Other parameters used the package default settings. Therefore, we had 15 different combinations of *mtry* and *ntree*.

In addition, we performed a 10-fold CV. The original dataset was partitioned into 10 equal subsets, and for each of the 10 iterations, one subset was picked as the testing data and the rest nine subsets served as the training data. Thus, each sample was used in the testing data exactly once, and the CV accuracy was computed as the percentage of correctly classifying all the testing samples. For each parameter combination and each fold of the CV (150 iterations in total), we repeated the random forests algorithm 10 times given the stochastic nature of bootstrapping and selecting variables to grow the trees. Since each execution of the random forests will yield a unique predictive model, we computed the accuracy of classifying each testing sample as the fraction of it being correctly predicted by the 10 models resulted by the 10 executions of the algorithm. Then the *average CV accuracy* using a specific combination of parameters was computed as the averaged accuracy across all testing samples. The parameter combination with the best average CV accuracy will be picked.

When the best combination of parameters was picked, the predictive models learned using it were then used to rank features according to the importance of the features in classification. For random forests, we used Gini Index as the quantification of feature importance (Breiman, 2017). Gini Index measures the inequality of dividing samples of two classes using a feature at a node of the trees. A higher Gini Index of a feature infers a better ability of using this feature to differentiate the two classes of the samples. Since we had multiple learned predictive models as a result of CV and repetitive runs, the final importance score of a feature was computed as the average Gini Index across all models.

### Gradient boosting machine

Another method to generate an ensemble is *boosting* where the base learners evolve over time and make weighted votes (Friedman, Hastie & Tibshirani, 2001). In a boosting algorithm, many base learners are built and the new learners improve on the previous ones. The learners are trained sequentially, which result in building a “committee” of complex predictors (Friedman, Hastie & Tibshirani, 2001; Friedman, 2001).

Gradient boosting machine is a boosting machine learning algorithm in which a weighted combination of predictors are used to make the final prediction (Friedman, 2001). A set of *n.trees* base learner trees are created in an iterative fashion, where a new one learns from the previous one. Let *F_m_*(***x***), where *m* = 1, 2,…,*n.trees*, denote the approximation that maps the input variables ***x*** to the desired output *y*. At iteration *m*, a new approximation *F_m_*(***x***) is constructed through improving on the previous one *F_m−_*_1_(***x***) by adjusting it using the gradient of the loss function ∇*L*(*y*, *F_m−_*_1_(***x***)).

For implementation, we used an R package called “gbm” (Ridgeway, 2007). Similar to random forests, we chose a range of values for each hyper-parameter in order to search for the best combination. GBM has three main parameters, *n.trees* (the number of trees), *interaction.depth* (the complexity of interactions between nodes, i.e., features), and *shrinkage* (the learning rate or step-size reduction). The testing values of these parameters were as follows: *n.trees* = {100, 500, 1,000, 2,000}, *interaction.depth* = {1, 2, 10}, and *shrinkage* = {0.001, 0.01, 0.1}. Therefore, we had 36 different combinations of the three parameters. Other parameters of GBM, such as *n.minobsinnode* (minimum number of samples in the tree terminal nodes), *bag.fraction* (the fraction of the training set samples randomly selected to construct a new tree), and *train.fraction* (the fraction of samples used to fit a GBM model), were set to default values as 10, 0.5, and 0.5, respectively.

Similar to random forests implementation, we used a 10-fold CV and repeated the algorithm 10 times for each combination of parameters and each fold of the CV. The best combination of parameters found was then used to rank the features. Feature Importance was estimated based on the number of times a feature is selected for splitting and the improvement to the model as a result of each split, weighted by the number of observations each split operates, and averaged over all trees (Friedman, Hastie & Tibshirani, 2001). Again, since we repeated GBM algorithm 10 times for each iteration of the 10-fold CV, the final importance score of a feature was averaged over 10 runs and 10-folds.

### Statistical interaction analysis

Both the random forests and GBM algorithms provide importance estimations on each SNP based on their contribution to the accurate classification. Such importance scores by the ensemble algorithms help to identify the top-ranked SNPs that are most possibly associated with the disease of CRC. It has been suggested that tree-based ensemble learning algorithms are able to detect interaction effects among multiple SNP features. Therefore, we employ an information gain method (Hu et al., 2011; Hu et al., 2013a, 2013b) to quantitatively investigate the statistical evidence of pairwise and three-way interactions among the top identified SNPs.

The information gain method considers the genotype of each SNP and the disease outcome as random variables. It first computes the mutual information *I*(*A*; *D*) between SNP *A* and the disease outcome *D* as the main effect of the individual SNP *A*, since it captures how much information of the disease status the genotype of SNP *A* provides. Then the information gain *IG*(*A*; *B*; *D*) of combining two SNPs *A* and *B* on explaining the disease status *D* is computed by subtracting the main effects of *A* and *B*, that is, *I*(*A*; *D*) and *I*(*B*; *D*) from the total mutual information *I*(*A*; *B*; *D*) by joining *A* and *B* to explain *D*. Essentially, the information gain *IG*(*A*; *B*; *D*) captures the *gained* information of combining *A* and *B* on explaining *D*, that is, the synergistic and non-additive interaction effect between SNPs *A* and *B*.

Similarly, when three SNPs, *A*, *B*, and *C*, are considered, the information gain *IG*(*A*; *B*; *C*; *D*) is computed by subtracting all the main effects and pairwise interaction effects from the total mutual information *I*(*A*; *B*; *C*; *D*) between joining *A*, *B*, and *C* together and the disease status *D*. Such an information gain metric measures the pure three-way synergistic interaction among three SNPs on explaining the disease outcome.

Permutation testing can be used to assess the significance level of a computed information gain value. In each permutation, the disease status labels of the data are randomly shuffled, and the information gain values are computed again for the permuted data. In this study, we performed a 1,000-fold permutation testing, that is, 1,000 permutations were collected to provide a null distribution of the assumption that there was no association between the genotypes and the disease outcome. Then the *p*-value was assessed for each computed information gain by counting the portion of permuted datasets that had a greater value of the information gain than the real data.

### Functional enrichment analysis

We also annotated the identified top SNPs with functional information using online resources including the ENSEMBL (http://www.ensembl.org) and the National Center for Biotechnology Information (NCBI) (https://www.ncbi.nlm.nih.gov/) databases. The databases provide biological information on the allele, chromosome, and gene information for each SNP.

Then we used the Database for Annotation, Visualization, and Integrated Discovery (DAVID) bioinformatics tool (Dennis et al., 2003; Huang, Sherman & Lempicki, 2008) to perform a functional enrichment analysis using the genes from the identified top SNPs. Functional enrichment analysis is a method to identify gene functional categories or associations with diseases that are over-represented in a set of genes. We compared the set of identified genes to the background of the entire human genome, in order to test which functional categories were significantly over-represented. The enriched functional categories may help better understand the disease of CRC. DAVID is widely used for enrichment analysis, specifically for exploring the functions of genes. It is a web-based tool which takes a list of genes as an input and produces annotation tables and charts to show diseases and relevant gene ontology (GO) terms enriched by a given list of genes.

## Results

In this section, we first show the result of feature selection and then the application results of the two ensemble learning methods on the CRC GWAS dataset. We performed the parameter tuning and used the parameter combinations of the random forest and the GBM algorithms that yielded the best classification accuracies. Meanwhile, both algorithms can rank the SNP variables based on their contributions to the classification. We identified the set of the best-ranked SNPs by both algorithms as our key possible susceptibility genetic markers, which were further investigated and validated through gene-gene interaction and pathway enrichment analyses.

### Filtered SNPs using TuRF

**Figure 1:**
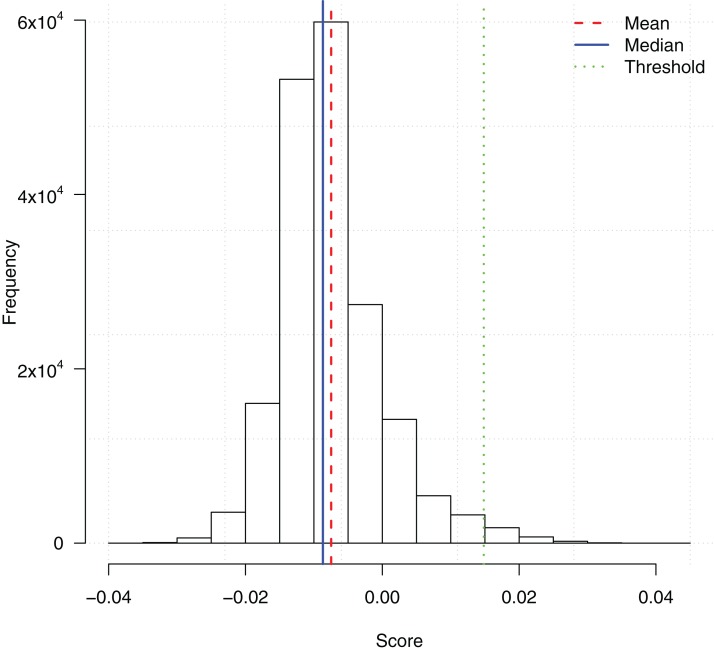
Histogram of SNP importance scores computed by the TuRF feature selection method. A cutoff threshold *mean* + 3*SD* is used to select the subset of the top 2,798 SNPs for the downstream ensemble learning analysis.

We ran the TuRF feature selection method on all 186,251 SNPs. Each SNP was assigned a score based on its contribution to the disease status. Since TuRF is a multi-variable feature selection algorithm, such a contribution may include both the individual main effect or the effect of interacting with other SNPs. Fig. 1 shows the distribution of the TuRF scores for all the SNPs.

To reduce the computational overhead of the downstream ensemble learning analysis, we then picked a score threshold and only included the potential SNPs with TuRF importance scores higher than the threshold. The threshold *mean* + 3*SD* was chosen which filtered the top 2,798 SNPs. The feature subset was an appropriate size such that the training of the ensemble learning algorithms could be finished in a reasonable time frame, meanwhile, enough features were included in the subsequent analysis to identify possible gene-gene interactions.

### Optimized parameters for the ensemble learning algorithms

The parameter tuning for the random forests algorithm included adjusting the values of two parameters, *mtry* = {100, 200, 300, 500, 1,000} and *ntree* = {500, 1,000, 2,000}. Thus, there were 15 combinations of parameter values. Recall that we ran the algorithm 100 times for each combination, the average CV accuracy and area under the curve (AUC) were computed over 100 runs.

**Figure 2:**
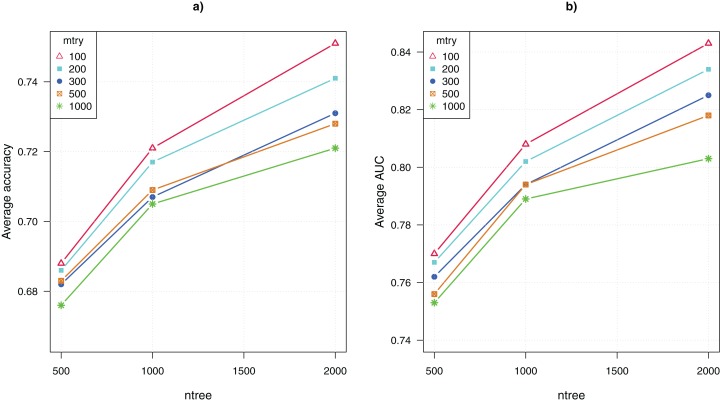
Parameter tuning for random forests. (A) Average CV accuracy using different parameter configurations. (B) Average CV area under the curve (AUC).

Figure 2 shows the comparative results for the 15 configurations of the random forests algorithm. The average CV accuracy and AUC are shown as a function of the parameter *ntree*, and different curves represent using different values of the other parameter *mtry*. The highest CV accuracy of 75% and AUC of 0.84 were achieved when *mtry* = 100 and *ntree* = 2,000. That is, the algorithm performed the best when *ntree* was set to the maximum value and *mtry* was set to the minimum value. This suggests that greater values of *ntree* and lower values of *mtry* are preferable for analyzing GWAS data. Both increasing the number of trees and reducing the number of randomly picked variables to construct each node of a tree enhance the diversity of trees in the ensemble. The result suggests that the genetic explanation of complex diseases may be highly heterogeneous, that is, a large number of diverse classification models that use different combinations of SNP attributes are needed to approximate the mapping from the genotypes to the disease outcome.

The GBM algorithm had three parameters, that is, *n.trees* = {100, 500, 1,000, 2,000}, *interaction.depth* = {1, 2, 10}, and *shrinkage* = {0.001, 0.01, 0.1}. Again, for each of the 36 combinations of parameter combinations, 10 runs were collected for each of the 10-fold CV, and the average CV accuracy and AUC were computed over 100 runs.

**Figure 3:**
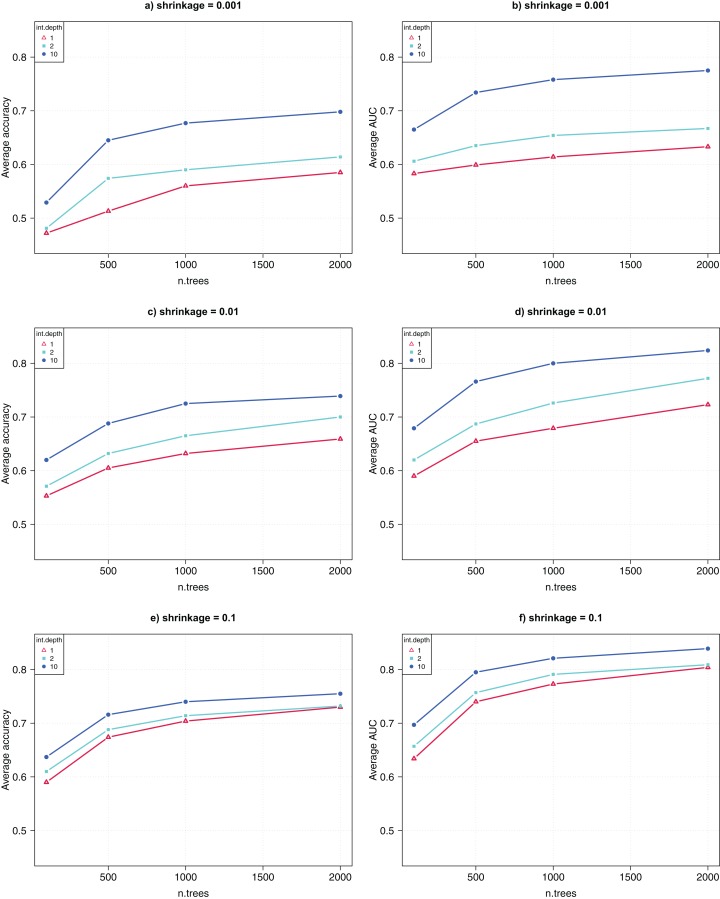
Parameter tuning for GBM. (A, C, E) show the average CV accuracy for *shrinkage* of 0.001, 0.01, and 0.1. (B, D, F) show the average CV AUC for *shrinkage* of 0.001, 0.01, and 0.1, respectively.

Figure 3 shows the comparative results for the parameter tuning. Each sub-figure uses a *shrinkage* value, and shows the average CV accuracy (left) and AUC (right) as a function of the parameter *n.trees*. The highest CV accuracy was 74% and the CV AUC was 0.82, achieved when *n.trees* was set to 2,000, *interaction.depth* was 10, and *shrinkage* was 0.1. This suggests that, for GWAS data analysis, GBM performs better when greater values of *interaction.depth*, *n.trees*, and *shrinkage* are used.

### Top-ranked SNPs

After parameter tuning, we chose the optimal parameter combinations of the random forests algorithm, that is, *mtry* = 100 and *ntree* = 2,000, and of the GBM algorithm, that is, *n.trees* = 2,000, *interaction.depth* = 10, and *shrinkage* = 0.1, to assess the importance of the SNP features. In random forests, the Gini Index importance of a feature was estimated based on the impurity of splitting testing samples on the tree node using the feature. Since we repeated the algorithm 100 times (10 times for each fold of the 10-fold CV), the final importance score (between 0 and 1) of a feature was computed as the averaged impurity score over 100 runs. The averaged importance score of each feature using GBM was computed similarly.

**Figure 4:**
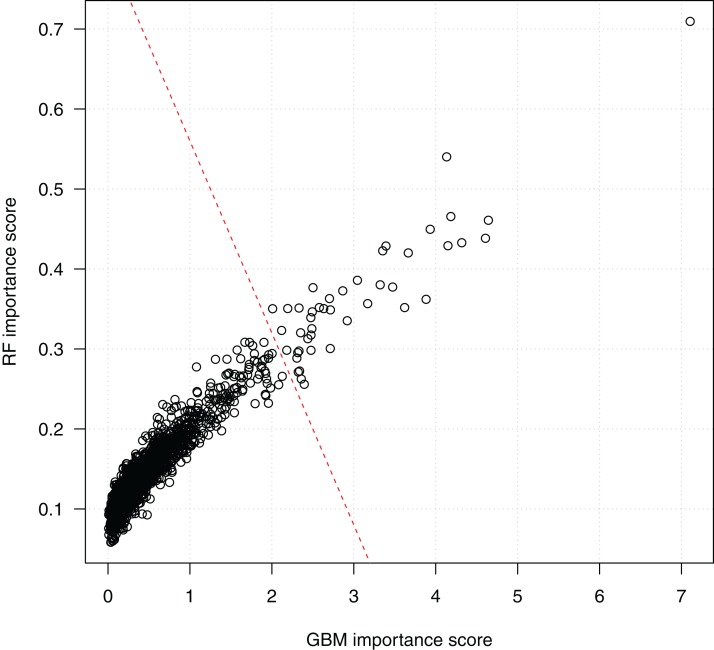
Scatter plot of SNP importance scores by the two ensemble learning algorithms. Each data point represents a SNP, and the *x*-axis shows its GBM importance score and the *y*-axis shows its random forests (RF) importance score. A separating line (red dashed) is used to show the most important SNPs identified by both algorithm.

Figure 4 shows the scores of the 2,798 SNPs assessed by both algorithms. We see that the scorings by random forests and GBM are highly correlated, which indicates the consistency of the two algorithms. Since a higher score indicates a higher importance of a SNP in classification, the top-right corner of the figure presents the SNPs ranked high by both the random forests and the GBM algorithms. The bottom-left corner of the figure includes a dense cluster of SNPs with low importance scores by both algorithms. We draw a line to separate SNPs into two clusters. The 44 SNPs on the right side of the separating line are the most important SNPs identified by both algorithms, and will be investigated subsequently on their biological functions and statistical interactions. Among them, SNP rs3760948 from gene *ARRDC5* is ranked as the most important by both algorithms.

**Figure 5:**
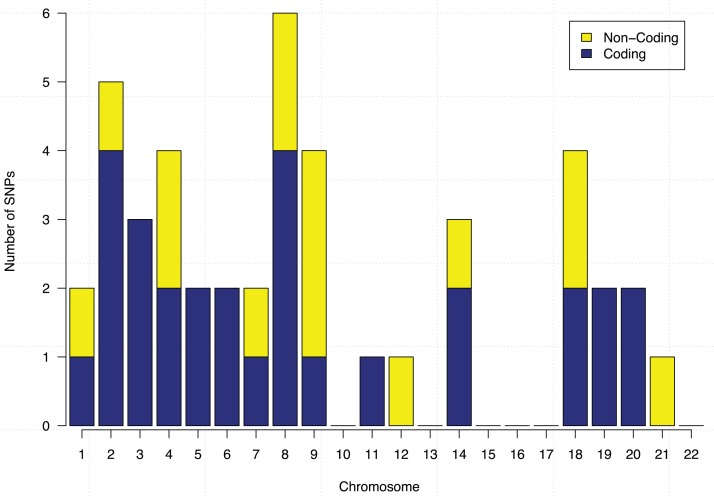
Chromosome distribution and the coding vs. non-coding regions of the identified 44 SNPs.

These 44 SNPs and their chromosome and gene information are listed in Table 1. Gene information of 29 SNPs was found using the ENSEMBL and the NCBI databases. In the table, 15 SNPs without any gene information are from non-coding DNA regions. The *p*-values of SNPs and their ranking among all 186,251 SNPs are also shown in the table. Figure 5 further shows the distribution of the 44 identified SNPs in each chromosome. The comparison of coding vs. non-coding regions is also shown. There were no identified SNPs in chromosomes {10, 13, 15, 16, 17, 22}. Chromosome eight contains six identified SNPs in which four of them belong to coding regions. Chromosome two has four out of five identified SNPs from the coding regions of DNA.

**Table 1:** List of the 44 identified top ranking SNPs.

Chromosome	SNP	A1	MAF	*p*-value	*p*-value rank	Gene
1	rs12407198	G	0.338	5.675 × 10^−3^	1,128	*C1orf101*
1	rs647831	G	0.345	1.041 × 10^−2^	2,018	–
2	rs1367128	G	0.192	3.527 × 10^−3^	724	*THSD7B*
3	rs1505229	T	0.391	9.559 × 10^−4^	208	*LRRTM4*
2	rs1816647	T	0.391	3.521 × 10^−2^	6,803	–
2	rs7594717	G	0.335	4.632 × 10^−5^	10	*ALK*
2	rs9288684	T	0.078	1.041 × 10^−4^	25	*INPP5D*
3	rs11185516	A	0.427	6.691 × 10^−1^	125,071	*ZDHHC19*
3	rs12695485	T	0.111	2.966 × 10^−2^	5,777	*LOC107986044*
3	rs6782709	G	0.345	1.885 × 10^−2^	3,663	*LOC105374217*
4	rs10016091	G	0.464	2.475 × 10^−3^	514	*SCFD2*
4	rs1991915	T	0.366	6.061 × 10^−3^	1,213	*OTOP1*
4	rs2010907	G	0.276	8.263 × 10^−4^	167	–
4	rs2736486	C	0.326	1.471 × 10^−2^	2,888	–
5	rs2406370	G	0.436	1.630 × 10^−1^	31,039	*ITGA1*
5	rs9688110	A	0.356	9.197 × 10^−4^	198	*FAT2*
6	rs7747931	A	0.433	4.637 × 10^−2^	8,963	*E2F3*
6	rs952880	C	0.485	2.318 × 10^−1^	43,974	*KCNQ5*
7	rs17162736	A	0.140	1.466 × 10^−2^	2,880	*STEAP2-AS1*
7	rs17379465	A	0.316	1.253 × 10^−1^	23,999	–
8	rs11783793	T	0.417	6.035 × 10^−4^	118	–
8	rs11985944	T	0.269	1.981 × 10^−3^	432	–
8	rs13263313	T	0.347	8.509 × 10^−3^	1,670	*JPH1*
8	rs1495008	C	0.170	3.740 × 10^−3^	765	*LOC101929628*
8	rs17831158	A	0.390	1.942 × 10^−3^	417	*LINC00968*
8	rs721619	G	0.335	1.755 × 10^−1^	33,327	*EPHX2*
9	rs10814848	G	0.507	1.000 × 10^−3^	223	*GLIS3*
9	rs3912454	C	0.471	3.876 × 10^−2^	7,479	–
9	rs4625115	T	0.402	6.728 × 10^−4^	138	–
9	rs4961513	A	0.291	3.435 × 10^−4^	62	–
11	rs6578849	G	0.379	5.830 × 10^−4^	111	*SYT9*
12	rs11610311	C	0.265	2.581 × 10^−3^	528	–
14	rs1212694	A	0.234	1.030 × 10^−3^	225	*ACTR10*
14	rs2645737	C	0.450	1.135 × 10^−2^	2,223	*NID2*
14	rs8022574	A	0.406	2.786 × 10^−3^	585	–
18	rs2571219	G	0.387	1.339 × 10^−3^	288	*ATP8B1*
18	rs3844138	A	0.250	1.120 × 10^−2^	2,193	–
18	rs658836	C	0.211	4.416 × 10^−4^	82	–
18	rs898438	G	0.366	9.927 × 10^−4^	218	*DCC*
19	rs344570	T	0.089	2.718 × 10^−4^	53	*TNFSF14*
19	rs3760948	T	0.371	2.007 × 10^−4^	46	*ARRDC5*
20	rs2179321	T	0.512	4.872 × 10^−2^	9,380	*PLCB4*
20	rs2386946	A	0.210	5.192 × 10^−3^	1,035	*CDH4*
21	rs3842986	T	0.224	4.063 × 10^−2^	7,875	–

**Note:**

A1 is the minor allele of a SNP, and MAF stands for the minor allele frequency. The *p*-value shows the confidence of a SNP’s association with the disease, computed using PLINK, as well as how it ranks among all 186,251 SNPs in the dataset (from the most to the least significant).

### Statistical interactions among the identified SNPs

We calculated the information gain between all $\left( \matrix{44 \hfill \cr 2 \hfill \cr} \right)$ pairs of the 44 identified SNPs. A 1,000-fold permutation test was performed to assess the significance level of each pair. Table 2 lists the 17 most significant SNP pairs (*p* < 0.02) with their information gain measures and the *p*-values. The maximum information gain was 1.3% for the interaction between SNPs rs2010907 and rs3760948 (*p* = 0.002), meaning that the synergistic interaction effect between the two SNPs explained 1.3% of the disease outcome. Six pairs of coding SNPs, that is, SNPs from genes, had significant interaction effects.

**Table 2:** Most significant pairwise interactions of the 44 identified SNPs.

SNP_1_ (gene_1_)	SNP_2_ (gene_2_)	Information gain (%)	*p*-value
rs2010907	rs3760948 (*ARRDC5*)	1.30	0.002
rs11185516 (*ZDHHC19*)	rs344570 (*TNFSF14*)	1.15	0.008
rs9688110 (*FAT2*)	rs658836	1.12	0.005
rs4625115	rs344570 (*TNFSF14*)	1.07	0.004
rs9288684 (*INPP5D*)	rs2179321 (*PLCB4*)	1.04	0.015
rs1367128 (*THSD7B*)	rs8022574	1.02	0.016
rs11185516 (*ZDHHC19*)	rs3842986	1.01	0.010
rs10814848 (*GLIS3*)	rs6578849 (*SYT9*)	0.98	0.008
rs1505229 (*LRRTM4*)	rs952880 (*KCNQ5*)	0.98	0.011
rs1505229 (*LRRTM4*)	rs11610311	0.98	0.013
rs9288684 (*INPP5D*)	rs2386946 (*CDH4*)	0.96	0.018
rs1991915 (*OTOP1*)	rs3842986	0.95	0.019
rs11783793	rs11610311	0.93	0.012
rs721619 (*EPHX2*)	rs4961513	0.92	0.017
rs4625115	rs2571219 (*ATP8B1*)	0.91	0.017
rs12695485 (*LOC107986044*)	rs898438 (*DCC*)	0.79	0.016
rs9288684 (*INPP5D*)	rs11985944	0.70	0.015

We then calculated the three-way information gain among all $\left( \matrix{44 \hfill \cr 3 \hfill \cr} \right)$ possible trios of the 44 identified SNPs. Table 3 lists the 16 most significant (*p ≤* 0.001) SNP trios with their information gain measures and *p*-values. The strongest three-interaction was found among SNPs from genes *OTOP1*, *EPHX2*, and *LINC* with a strength of 2.55% and a significance level of *p* < 0.001, suggesting that the pure three-way synergistic effect among the three SNPs can explain 2.55% of the disease outcome. There were six trios of significant interacting SNPs all from coding regions of DNA.

**Table 3:** Most significant three-way interactions of the 44 identified SNPs.

SNP_1_ (gene_1_)	SNP_2_ (gene_2_)	SNP_3_ (gene_3_)	Information gain (%)	*p*-value
rs1991915 (*OTOP1*)	rs721619 (*EPHX2*)	rs17831158 (*LINC*)	2.55	<0.001
rs1816647	rs10814848 (*GLIS3*)	rs3760948 (*ARRDC5*)	2.43	<0.001
rs11185516 (*ZDHHC*)	rs11985944	rs3844138	2.41	<0.001
rs2010907	rs9688110 (*FAT2*)	rs4625115	2.27	<0.001
rs7594717 (*ALK*)	rs721619 (*EPHX2*)	rs3760948 (*ARRDC5*)	2.25	<0.001
rs12695485 (*LOC107*)	rs17831158 (*LINC*)	rs13263313 (*JPH1*)	2.25	<0.001
rs647831	rs2736486	rs952880 (*KCNQ5*)	2.23	<0.001
rs1991915 (*OTOP1*)	rs8022574	rs2571219 (*ATP8B1*)	2.22	<0.001
rs11185516 (*ZDHHC*)	rs2736486	rs10814848 (*GLIS3*)	2.11	<0.001
rs7594717 (*ALK*)	rs13263313 (*JPH1*)	rs898438 (*DCC*)	1.93	0.001
rs17379465	rs2645737 (*NID2*)	rs658836	1.90	0.001
rs1367128 (*THSD7B*)	rs17831158 (*LINC*)	rs2386946 (*CDH4*)	1.89	0.001
rs12407198 (*C1orf101*)	rs10016091 (*SCFD2*)	rs2010907	1.86	0.001
rs1816647	rs6782709 (*LOC105*)	rs4961513	1.82	0.001
rs4961513	rs11610311	rs3842986	1.82	0.001
rs1367128 (*THSD7B*)	rs6578849 (*SYT9*)	rs344570 (*TNFSF14*)	1.40	0.001

### Enriched gene functional terms

We submitted the identified 29 genes to the DAVID software, and chose categories of Disease, GO, and Pathway for a functional enrichment analysis. We set the gene count threshold as five, that is, only functional categories including more than 4 of the 29 identified genes were considered, and the significance cutoff as 0.05, that is, Fisher’s exact test *p* < 0.05. Table 4 lists the most significantly enriched functional categories. There were 16 enriched terms, four of which were diseases, and the rest were GO terms. The most significantly enriched term was the disease *tobacco use disorder* with 14 out of 29 genes in the category and a significance level of 3.9 × 10^−5^.

**Table 4:** Enriched gene ontology (GO) terms on the 29 identified genes.

Category	Term	Gene count	*p*-value
GAD_DISEASE	Tobacco use disorder	14	3.9 × 10^−5^
GAD_DISEASE_CLASS	Chemdependency	14	3.0 × 10^−4^
GAD_DISEASE_CLASS	Metabolic	15	3.6 × 10^−3^
GOTERM_MF_DIRECT	Calcium ion binding	5	6.1 × 10^−3^
GAD_DISEASE_CLASS	Cardiovascular	13	6.5 × 10^−3^
GOTERM_MF_FAT	Calcium ion binding	5	6.7 × 10^−3^
GOTERM_BP_FAT	Movement of cell	7	1.2 × 10^−2^
GOTERM_CC_DIRECT	Integral component of membrane	12	1.7 × 10^−2^
GOTERM_MF_FAT	Metal ion binding	10	2.0 × 10^−2^
GOTERM_BP_FAT	Neuron development	5	2.2 × 10^−2^
GOTERM_MF_FAT	Cation binding	10	2.2 × 10^−2^
GOTERM_BP_FAT	Iocomotion	6	2.5 × 10^−2^
GOTERM_MF_FAT	Ion binding	10	2.8 × 10^−2^
GOTERM_CC_DIRECT	Plasma membrane	10	3.1 × 10^−2^
GOTERM_BP_FAT	Cell migration	5	4.2 × 10^−2^
GOTERM_BP_FAT	Neuron differentiation	5	4.7 × 10^−2^

## Discussion

Identifying genetic markers associated with complex human diseases helps us better understand the disease etiology in order to better diagnose, treat, and even prevent diseases. Given the complexity of human diseases, especially cancers, the causing factors are more plausibly interactions among multiple genetic attributes instead of individual contributions. However, searching for combinations of attributes imposes a significant challenge for bioinformatics and genome-wide association research, since thousands to a million possible genetic attributes can be included for investigation.

Powerful machine learning algorithms have been used for mining high-volume data in fields such as engineering, finance, and social sciences, and have started to see applications in analyzing high-dimensional biomedical data as well.

In this article, we explored the application of two ensemble learning algorithms, random forests, and GBM, in identifying interacting genetic attributes associated with CRC. We studied a GWAS CRC dataset collected from the Canadian province of Newfoundland. We performed data preprocessing and filtering using the TuRF feature selection algorithm. By parameter tuning, we optimized the parameters for both random forests and GBM for GWAS data analyses. Both ensemble learning algorithms produced rankings on the importance of SNP contribution to the disease classification. By comparing the rankings provided by both algorithms, we identified a set of 44 top ranked SNPs from both coding and non-coding regions of DNA (Table 1). The coding SNPs mapped to 29 genes, which included both known CRC association genes: *DCC*, *ALK*, *ITGA1*, *E2F3*, and *NID2*, and unknown but potential CRC association genes.

We performed the statistical interaction analysis of the 44 identified SNPs and were able to validate strong and significant pairwise and three-way gene-gene interactions (Tables 2 and 3). These included a three-way interaction among *ALK*, *JPH1*, and *DCC*. In addition, functional enrichment analysis on the set of the 29 identified genes suggested 16 significantly enriched functional terms including four diseases: *tobacco use disorder*, *chemical dependency*, *metabolic diseases*, and *cardiovascular diseases*, and 12 GO terms (Table 4). We highlight some important findings in the following paragraphs.

SNP rs3760948 from gene arrestin domain-containing 5 (*ARRDC5*) was ranked the highest by both random forests and GBM (Fig. 4). It was detected with the strongest pairwise interaction with a non-coding SNP rs2010907 (Table 2) and the second strongest three-way interaction with SNP rs10814848 from gene GLIS family zinc finger 3 (*GLIS3*) and non-coding SNP rs1816647 (Table 3). This is a supportive evidence that both ensemble algorithms can detect interacting genetic attributes.

The gene “deleted in CRC” (*DCC*) is a tumor suppressor in CRC (Castets et al., 2012) and is well known to be associated with CRC (ENSEMBL database: http://www.ensembl.org/Homos_sapiens/Gene/Phenotype?db=core;g=ENSG00000187323). Anaplastic lymphoma kinase (*ALK*) gene is directly related to colorectal adenocarcinoma, and may affect treatments for advanced CRC (Lipson et al., 2012; Aisner et al., 2014; Pietrantonio et al., 2014). Genes *DCC* and *ALK*, along with junctophilin-1 *JPH1*, were found having a strong (1.93%) and significant (*p* = 0.001) three-way interaction. Gene α1-integrin (*ITGA1*) and transcription factor *E2F3* have been found related to the prevention of tumor progression in colon cancer patients (Van Slambrouck et al., 2007; Akao et al., 2011; Boudjadi et al., 2016). Gene nidogen-2 (*NID2*) has been found to be associated with lung cancer (Zhang et al., 2014).

Our results also suggested novel genes associated with the disease of CRC. For instance, enzyme gene phosphatidylinositol-3,4,5-trisphosphate 5-phosphatase 1 (*INPP5D*) had strong and significant pairwise interactions with another enzyme gene 1-Phosphatidylinositol-4,5-bisphosphate phosphodiesterase beta-4 (*PLCB4*) and gene cadherin-4 (*CDH4*), as well as a non-coding SNP rs11985944. We have not seen any published literature associating *INPP5D* with CRC and related diseases, and it can be a possible risk association gene to investigate in biology.

We found 14 genes associated with the functional term *tobacco use disorder* (*p* = 3.9 × 10^−5^) (Table 4). In a study on tobacco use and the risk of CRC using a retrospective cohort study of germ-line mutants, tobacco use was found to significantly increase the risk of CRC (Watson et al., 2004). Another significantly enriched functional term was *cell migration*, which has been reported to be related to cancers (Friedl & Wolf, 2003; Paul, Mistriotis & Konstantopoulos, 2017). The tumor cells migrate and enter the blood and will go to other tissues and spread cancer. When CRC cells spread, they most often spread to the liver. Our results suggest the possible importance of confining cell mobility in CRC treatment and prevention.

Our future studies include: (1) utilizing more computational power in order to include more SNP attributes in the classifier training and feature importance analysis and (2) exploring or combining other machine learning algorithms, including neural networks and evolutionary algorithms, for the search of gene-gene interactions.

## Conclusions

Machine learning algorithms have seen increasing applications in bioinformatics and computational biology thanks to their powerful abilities of automatic learning and modeling complex relationships among a large number of features. Applications of machine learning techniques require domain-specific tailoring and careful design since an application area has unique problem definitions and challenges. In the context of GWAS, we need algorithms that are able to detect the non-linear, non-additive interactions among multiple genetic factors that contribute to the disease outcome. This article demonstrates a novel design of an informatics framework of using two ensemble learning algorithms to search for interacting genetic factors associated with cancer and validating the results through statistical interaction analysis and biological functional enrichment analysis. With further biological experiments, our bioinformatics findings may help us better understand the disease etiology of CRC. We also hope our study can inspire more bioinformatics tool developments for human disease association studies.

## Additional Information and Declarations

### Data Availability

The following information was supplied regarding data availability: Colorectal Transdisciplinary (CORECT) Study: https://research.fhcrc.org/peters/en/corect-study.html GitHub: https://github.com/MIBlab-MUN/ensemble-learning-GWAS.git.
